# Edge-to-Edge Repair for Tricuspid Valve Regurgitation. Preliminary Echo-Data and Clinical Implications from the Tricuspid Regurgitation IMAging (TRIMA) Study

**DOI:** 10.3390/jcm11195609

**Published:** 2022-09-23

**Authors:** Myriam Carpenito, Valeria Cammalleri, Luka Vitez, Aurelio De Filippis, Edoardo Nobile, Maria Caterina Bono, Simona Mega, Matjaz Bunc, Francesco Grigioni, Gian Paolo Ussia

**Affiliations:** 1 Operative Research Unit of Cardiovascular Science, Fondazione Policlinico, Universitario Campus Bio-Medico, Via Alvaro del Portillo, 200, 00128 Roma, Italy; 2 Department of Cardiology, University Medical Center Ljubljana, 1000 Lubljana, Slovenia; 3 Research Unit of Cardiovascular Science, Department Medicine, Università Campus Bio-Medico di Roma, Via Alvaro del Portillo, 21, 00128 Roma, Italy

**Keywords:** tricuspid valve, tricuspid regurgitation, transcatheter interventions, edge-to-edge repair, echocardiography

## Abstract

Background: The natural history of tricuspid valve regurgitation (TR) is characterized by poor prognosis and high in-hospital mortality when treated with isolated surgery. We report the preliminary echocardiographic and procedural results of a prospective cohort of symptomatic patients with high to prohibitive surgical risk and at least severe TR who underwent transcatheter edge-to-edge repair through the TriClip^TM^ system. Methods: From June 2020 to March 2022, 27 consecutive patients were screened, and 13 underwent transcatheter TriClip^TM^ repair. In-hospital, 30-day and six-month clinical and echocardiographic outcomes were collected. Results: Nine patients had severe, three massive and one baseline torrential TR. Sustained TR reduction of ≥1 grade was achieved in all patients, of which 90% reached a moderate TR or less. On transthoracic echocardiographic examination, there were significant reductions in vena contracta width (*p* < 0.001), effective regurgitant orifice area (*p* < 0.001) and regurgitant volume (*p* < 0.001) between baseline and hospital discharge. We also observed a significant reduction in tricuspid annulus diameter (*p* < 0.001), right ventricular basal diameter (*p* = 0.001) and right atrial area (*p* = 0.026). Conclusion: Treatment with the edge-to-edge TriClip device is safe and effective. The resulting echocardiographic improvements indicate tricuspid valve leaflet approximation does not just significantly reduce the grade of TR but also affects adjacent structures and improves right ventricular afterload adaptation.

## 1. Introduction

Moderate or severe tricuspid regurgitation (TR) is a valvular pathology associated with considerable prevalence (0.55%) and increased morbidity and mortality in the general population [1]. The aetiology is mostly secondary, arising as a result of chronic atrial fibrillation and left-sided valvular or myocardial dysfunction and subsequent right ventricular pressure and/or volume overload. This can further lead to right-sided heart failure, increased hospitalisations and poor prognosis [2]. Even though isolated tricuspid valve (TV) surgery is the recommended treatment in carefully selected candidates [3], previous studies showed poor outcomes and high in-hospital mortality [4,5,6]. With the advent of various percutaneous techniques and devices for the treatment of TR [7], transcatheter edge-to-edge repair (TEER) has shown promising results with marked clinical and echocardiographic benefits as well as favourable one-year survival [8,9]. However, the patient selection process remains challenging in this study population due to a lack of guidelines, clear selection criteria and late patient referral to tertiary centres [10,11,12]. We aimed to report the preliminary echocardiographic and procedural results of a prospective cohort of “real-life” symptomatic patients with high to prohibitive surgical risk, impaired right ventricular (RV) function and at least severe TR who underwent transcatheter tricuspid edge-to-edge repair through the TriClip^TM^ system (Abbott Vascular, Santa Clara, CA, USA).

## 2. Materials and Methods

### 2.1. Study Population

This prospective, single-arm study, conducted at the University Campus Bio-Medico of Rome, Italy, screened 27 consecutive symptomatic patients referred with the diagnosis of at least severe TR from June 2020 to March 2022. The anatomical feasibility was established according to a detailed transoesophageal echocardiogram (TEE) and a dedicated computerised tomography (CT) for the right cardiac chamber [13]. Patients with the following criteria were included in the study: symptomatic heart failure, at least severe TR, adequate transoesophageal windows, suitable anatomy for leaflet grasping (sufficient tissue for grasping, leaflet lengths < 7 mm and primary TR with prolapse or secondary TR with normal appearing leaflet mobility), normal to moderately reduced RV function, systolic PAP < 60 mmHg, absence of significant valvular calcifications and active malignancy. Exclusion criteria were as follows: non-suitable anatomy for leaflet grasping (severe leaflet thickening or shortening, insufficient echocardiographic windows for leaflet visualisations, complex TV morphology including four-leaflet configuration), severely reduced RV function, systolic PAP > 60 mmHg, prior TV surgery, congenital heart disease requiring surgical intervention, active endocarditis and participants requiring emergent cardiac surgery. This study complies with the Declaration of Helsinki and was approved by the local ethic committee (103/21 OSS ComEt CBM). All patients provided written informed consent.

### 2.2. Echocardiography

All patients underwent pre-procedural planning with transthoracic echocardiogram (TTE) and TEE followed by a dedicated CT scan. The transgastric short-axis view obtained with TEE allowed us to analyse the origin of TR jet and plan an adequate grasping strategy. Additional helpful information was obtained from the 4-chamber, alternative 4-chamber and deep/mid oesophageal right ventricle inflow/outflow views in multiplane collected using Siemens Acuson SC2000 (Siemens Medical Systems, Pleasanton, CA, USA). The grasping strategy was planned on screening images analysed by a committee of echocardiographers and interventional cardiologists by choosing which leaflets to grasp, in which order and at which distance from the commissures. All TTE data were collected using a GE Vivid E95 (Boston, MA, USA) echocardiography machine. The attending physician manages the volemic status. Standard parameters were recorded according to the American Society for Echocardiography (ASE) and European Association of Cardiovascular Imaging (EACVI) guidelines [14,15]. TR and right ventricular function were assessed by quantitative, semiquantitative and qualitative parameters: right ventricular chamber and annulus dimensions, tricuspid annular plane systolic excursion (TAPSE), effective regurgitant orifice area (EROA), regurgitant volume, vena contracta width (VC) and colour flow Doppler. Coupling between the right ventricle and pulmonary artery was calculated through the ratio between TAPSE and echocardiographically estimated pulmonary artery systolic pressure (PASP). TR was assessed by standard 2D colour Doppler methods and classified using a prespecified 5-class scheme: mild, moderate, severe, massive and torrential [16]. This extended classification scheme was used to detect subtle changes even when moderate TR or less was not achieved.

### 2.3. Procedure

Transcatheter edge-to-edge repair was performed with the TriClip^TM^ device (Abbott Vascular, Santa Clara, CA, USA) via right common femoral vein access. Afterwards, a 24 F steerable guiding catheter was placed in the right atrium (RA). Under fluoroscopic and TEE guidance, the TriClip delivery system was introduced in the right atrium and oriented perpendicular to the TV. Subsequently, the clip was opened and advanced in the RV. Once the correct orientation and position were obtained, the clip was pulled back to grasp the target leaflets. Following TEE control, the clip was released. One additional clip was placed in case of insufficient reduction of TR. Venous access was closed using a Perclose ProGlide^TM^ (Abbott Vascular, Santa Clara, CA, USA) suture device.

### 2.4. Outcomes and Endpoints

The primary efficacy endpoint was a TR reduction of at least one grade as assessed by transthoracic echocardiography (TTE) at discharge (procedural success). The primary safety endpoint was a composite of major adverse events (MAE) at one month, including cardiovascular mortality, myocardial infarction, new onset renal failure, stroke, endocarditis and non-elective cardiovascular surgery for TV repair system-related adverse events. Secondary endpoints were: acute implant success (defined as successful delivery and deployment of at least one clip to achieve leaflet approximation and retrieval of the delivery system) and in-hospital, 30-day and six-month clinical and echocardiographic outcomes. Clinical and functional status was assessed by the New York Heart Association (NYHA) class, and health-related quality of life was assessed with the EuroQol-5-dimension (EQ-5D) questionnaire by assigning a value from 1 to 5 for five domains: mobility, self-care, habitual activities, pain/discomfort and anxiety/depression.

Echocardiographic parameters evaluated during the scheduled follow-up time were: the TR grade, the device durability, the right chamber function and dimension, the inferior vena cava dimension and TAPSE/PAPS coupling.

All major adverse events and additional safety endpoints (major bleeding, new-onset liver failure, pulmonary thromboembolism, device embolisation, single leaflet device attachment, hospitalisations) were collected during the follow-up time at 30 days and six months.

### 2.5. Statistical Analysis

Longitudinal data for continuous variables were analysed using a generalised linear model with unstructured correlation to account for repeated measures. Data are presented as mean ± standard error for normally distributed or median and interquartile ranges for asymmetrically distributed continuous variables. Proportions are presented for categorical variables, and a *t*-test was used to compare paired nominal data. Paired two-samples Wilcoxon U test was used for not normally distributed data. Distribution was tested according to Shapiro–Wilk test. Differences were considered significant at *p* < 0.05. All statistical analyses were performed using the SPSS Statistics V23 software package (IBM, Armonk, NY, USA).

## 3. Results

### 3.1. Baseline Characteristics

Thirteen subjects (85% female) with significant comorbidities and high surgical risk were included. The mean age was 81 ± 4 years, with an average EuroSCORE II of 8 ± 4% (Table 1). TR included functional (77%) and CIED-induced TR (23%) [17]. The most common comorbidities were atrial fibrillation (100%), hypertension (92%), diabetes (31%) and prior coronary artery disease (31%). Two patients had a previous surgical mitral intervention, while three patients underwent a TAVI procedure. All patients were classified as at least NYHA functional class III. Nine patients (69%) had severe, three patients (23%) massive and one patient (8%) torrential TR. In-hospital and 30-day clinical and echocardiographic outcomes were recorded for all patients. Six-month TTE follow-up to assess device durability and TR grade was carried out in 10 of 13 patients (77%) since the procedure was performed less than three months ago in the last three patients.

### 3.2. Procedural and Echocardiographic Results

The mean device time was 49 ± 25 min, and the mean procedural time was 88 ± 31 min. The primary efficacy endpoint (procedural success) and the acute implant success were achieved in all cases, implanting one device in eight patients (62%) and two in five patients (38%). The device was placed antero-septal in 85% and postero-septal in 46% of cases. Immediately after the procedure, we observed a significant improvement in TR grade: six (46%) subjects had moderate, six (46%) mild and one patient (8%) with previous torrential TR treated with two clips had severe post-procedural TR because of partial leaflet detachment 48 h later. All patients were extubated in the catheterisation laboratory. There were no procedural or in-hospital MAEs.

**Figure 1:**
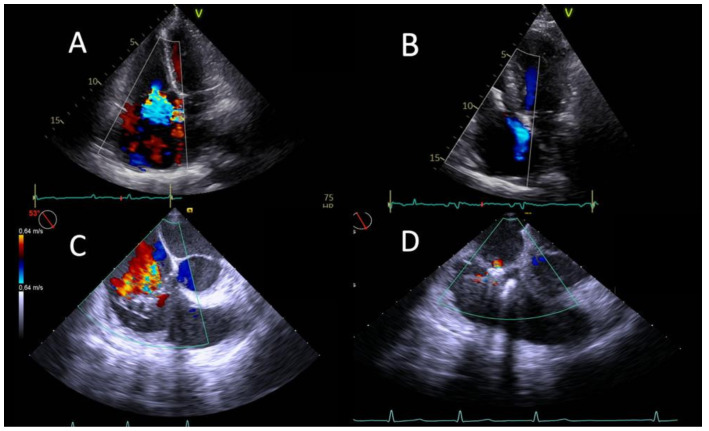
Echocardiographic pictures of successful TR reduction after TEER. Transthoracic echocardiographic four-chamber view at baseline (**A**) and at discharge (**B**) after implantation of one TriClip XT. Transoesophageal echocardiographic right ventricle inflow/outflow view showing severe tricuspid regurgitation mainly originating from septal and anterior leaflet at baseline (**C**) and traces of regurgitation after implantation of one device (**D**).

On echocardiography, significant reductions in VC width (8 ± 1 to 4 ± 2 mm; *p* < 0.001), EROA (0.63 ± 0.28 to 0.32 ± 0.21 cm^2^; *p* < 0.001) and regurgitant volume (57 ± 16 to 28 ± 16 mL/beat; *p* < 0.001) occurred between baseline and before hospital discharge. We also observed a change in adjacent anatomical structures with a significant reduction in tricuspid annulus diameter (44 ± 5 to 40 ± 4 mm; *p* < 0.001), right ventricular basal diameter (47 ± 7 to 43 ± 4 mm; *p* = 0.001), right atrial area (27.8 ± 8 to 25.9 ± 8.3 cm^2^; *p* = 0.026) and inferior vena cava diameter (22 ± 3 to 18 ± 5 mm; *p* = 0.004). While three patients demonstrated a reduced TAPSE/PASP ratio (<0.31 mm/mmHg) before the intervention, the overall ratio significantly improved after device placement (0.37 ± 0.1 to 0.46 ± 0.1 mm/mmHg; *p* = 0.011) (Table 2). Importantly, the reduction in TR grade achieved before discharge was durable at short-term (30-days) and at the 6-month follow-up. All patients (100%) experienced a sustained TR reduction of ≥1 grade at six months, of which 90% (9 of 10) achieved a moderate TR or less (Figure 1).

### 3.3. Clinical Outcomes

The primary safety endpoint was achieved in all cases. Specifically, no cases of cardiovascular death, myocardial infarction, renal failure, stroke, endocarditis, and non-elective cardiovascular surgery occurred at one month. 

Sustained improvements were seen in clinical status, quality of life and hospitalisation rates between the 30-day and 6-month follow-up. Clinical improvements mainly occurred within the first month post-procedure, with all patients reaching NYHA class II or less (3.1 ± 0.4 vs. 1.9 ± 0.3, *p* < 0.001) and showing marked benefit in quality of life (0.58 ± 0.3 vs. 0.87 ± 0.4, *p* = 0.04). The overall mortality was 0%, with no subjects experiencing MAEs during a mean follow-up of six months. Single leaflet clip detachment occurred in one patient in whom two clips were implanted 48 h post-procedure and resulted in a reduction from torrential to severe TR grade. There were no cases of re-hospitalisation, major bleeding, pulmonary thromboembolism, new-onset of liver failure or device embolisation (Table 3).

## 4. Discussion

In this study, we report the initial experience and clinical results of the relatively novel treatment strategy of TEER in patients with isolated TR with high surgical risk, deemed inoperable with impaired right ventricular function. The main findings are: (1) TEER is effective and durable in reducing TR; (2) positive structural right chamber reverse remodelling occurs soon after the procedure; and (3) sustained improvements in clinical status, quality of life and no evidence of re-hospitalisation up to six months were seen in a high-risk patient cohort with no MAE. 

TR correction timing is critical and particularly challenging. It requires a multimodality imaging approach to confidently determine the aetiology and the severity of the TV lesion, the extent of remodelling and the functional status of ventricles. The difficulty in determining the right moment to perform the valvular repair arises from the fact that the severity of TR is highly dependent on the stress situation and may vary depending on the time of examination in the same patient. As more left valve diseases are treated with transcatheter therapies and evaluating the negative impact of TR on survival, the importance of developing transcatheter solutions for this subset of patients has recently been highlighted [18,19]. Although initially promising, most transcatheter-based TV repair techniques are still in development, and data on acute or prolonged follow-up are lacking. Currently, the most commonly used technique is the edge-to-edge repair of the TV, which acts on the tricuspid leaflets to improve the coaptation of the regurgitant valve. As demonstrated in previous studies, the procedural success, defined as effective clip placement and reduction in TR grade for ≥1 on TTE at the 30-day follow-up was the only predictor for reduced mortality and heart failure hospitalisation [9,20]. In addition, outcomes following tricuspid TEER have been strongly correlated with RV systolic function [21,22]. Remodelling of the RV was observed in patients after tricuspid TEER [9]; however, the extent of remodelling and the relationship to baseline measurements are currently not completely understood.

The epidemiological data from our cohort suggests that TR is predominantly functional due to enlargement of the tricuspid annulus and leaflet tethering, to a lesser extent, by the interference of the movement of the valve leaflets from the ventricular electrodes. However, the proportion of CIED-induced TR is expected to increase due to the ageing population and increased number of implantations. The pathophysiological relationship between the presence of the electrodes and the occurrence of significant TR or exacerbation of a pre-existing disease is a relatively emergent clinical challenge [17]. 

Regardless of aetiology, among our results, the observed TR reduction, device durability and lack of major clinical complications are likely the essential findings in agreement with previous data on the effects of TR reduction on clinical outcomes [9,20,23,24,25].

The primary efficacy endpoint was achieved in 100% of patients. Moreover, no further subjects experienced a reduction in TR grade between the 30-day and 6-month follow-ups. In one patient who had a baseline torrential TR, a reduction to severe grade was observed, without a negative impact on the clinical outcome. In the TRILUMINATE trial, the persistence of severe or more TR after TEER was associated with increased mortality and hospitalisations for heart failure at 1 year. Despite the limited follow-up time, clinical improvement occurred in most patients, as evidenced by a NYHA functional class ≤ II at 6 months in all patients. The present results are consistent with those observed in the TRILUMINATE trial at short- and long-term follow-up [9,23].

In our study, the reverse remodelling appeared to occur early after the procedure. In the TRILUMINATE trial, a reduction in RA and RV volumes was observed at 30 days and persisted at one year, with a progressive improvement in tricuspid annular plane systolic excursion (TAPSE) within the first year. In our study, a straight-line reduction in right ventricular dimension was observed in the acute phase after TEER, attributable to an acute change in RV loading condition. These results are not followed by an increase in right ventricular function immediately after the procedure, which is consistent with previous studies [23]. Interestingly, there is no evidence of further structural or functional improvements during the 6-month follow-up period beyond the short-term changes, according to the previous data [26]. This may be because TEER was performed too late in some patients when a consequence of chronic ventricular volume overload could become irreversible. Accordingly, we had three patients with a TAPSE/PASP ratio < 0.31 mm/mmHg at the time of TR diagnosis which is known to be independently connected with poor prognosis [27]. The ratio represents a robust hemodynamic marker associated with all-cause mortality in patients undergoing TV TEER [28]. An increase in the RV-PA coupling ratio may indicate an adequate ventricular and arterial response to TR reduction [29]. Reverse right ventricular remodelling results from reduced right ventricular size and increased functional indices, most likely due to the long-term effects of preload reduction after the initial period of afterload increase. This is consistent with previous analysis, where a lower TAPSE/PASP ratio was associated with advanced right heart remodelling, secondary TR and impaired functional capacity [30,31].

Among our patients with a baseline TAPSE/PASP ratio < 0.31, we reported a high prevalence of heart failure symptoms and comorbidities, with more severe TR and advanced RV remodelling. The intervention aimed at reducing RV pressure and volume overload could benefit RV systolic function and improve RV−PA coupling and patient prognosis [27]. This was in line with our cohort in which the overall ratio improved significantly after device placement, with no MAEs. Furthermore, despite the significant increase in post-procedural mean transvalvular gradient compared to baseline, it was found to have no clinical impact, as demonstrated by the improvement in clinical status and quality of life.

Our preliminary study in patients with more than severe TR showed that TEER is a feasible, safe and effective procedure with improved short-term echocardiographic parameters and outcomes. Marked changes were not just related to the reduction of TR but also to adjacent anatomical structures, including the tricuspid annulus, right atrium, right ventricle, and inferior vena cava. Effective grasping of the leaflet was shown to improve right ventricular afterload adaptation. In addition, it should be emphasized that one-year follow-ups are underway, which may provide encouraging data on late changes in right ventricular function.

The main limitation of this study is the single-site data collection and the small sample size. Due to the small sample size, we cannot draw meaningful statistical conclusions and consider it a preliminary and hypothesis-generating study. Patients and their anatomy were evaluated at enrolment by a committee focused on morphologic suitability for implantation of the device in the TV. In addition, other factors such as imaging views, the coaptation gap, the presence of leads across the TV and their interaction with the valve were also considered. Therefore, the results of this early feasibility study may not be generalizable to the overall population of patients with significant TR. In addition, the sample size may not be sufficient to identify predictors of improved NYHA functional class or echocardiographic results. An important limitation of our study is that it did not provide a long-term follow-up, but the results were very encouraging, even in the short term. Our future research goal is to refine and expand our dataset and continue patient follow-ups for at least one year to overcome these limitations.

## 5. Conclusions

In this single-centre experience, we have shown that treatment with the edge-to-edge TriClip device is safe and effective in patients with significant TR and is associated with marked clinical benefits. The resulting echocardiographic improvements indicate tricuspid valve leaflet approximation does not just significantly reduce the grade of TR, but also affects adjacent structures and improves right ventricular afterload adaptation.

## Figures and Tables

**Table 1:** Baseline clinical characteristics.

Patient Characteristics	N = 13
Mean age, years ± SD	81 ± 4
Gender-female, *n* (%)	11 (85)
BSA, m^2^ ± SD	1.7 ± 0.2
STS score, % median (Q1–Q3)	5.8 (3.8–7.7)
EuroScore II, % ± SD	8 ± 4
Diabetes, *n* (%)	4 (31)
Hypertension, *n* (%)	12 (92)
Hyperlipidaemia, *n* (%)	5 (38)
Coronary artery disease, *n* (%)	4 (31)
Smoking, *n* (%)	1 (8)
Atrial fibrillation, *n* (%)	13 (100)
History of cerebrovascular insult, *n* (%)	2 (15)
COPD, *n* (%)	2 (15)
CIED, *n* (%)	4 (31)
Previous TAVI implantation, *n* (%)	3 (23)
NYHA functional class III or more, *n* (%)	13 (100)
**Medications**	
Salicylic Acid, *n* (%)	3 (23)
Novel oral anticoagulant, *n* (%)	8 (62)
Warfarin, *n* (%)	4 (31)
Angiotensin-converting enzyme inhibitor/angiotensin receptor blocker, *n* (%)	6 (46)
Digoxin, *n* (%)	3 (23)
Calcium channel blocker, *n* (%)	3 (23)
Beta-blocker, *n* (%)	11 (85)
Diuretic, *n* (%)	13 (100)

BSA: Body Surface Area; CIED: cardiac implantable electronic device; COPD: chronic obstructive pulmonary disease; NYHA: New York Heart Association; STS: Society of Thoracic Surgeons Score; TAVI: Transcatheter aortic valve implantation.

**Table 2:** Echocardiographic data of n.13 patients between baseline and before hospital discharge.

Echocardiographic Parameter	Pre-TriClip	Post-TriClip	*p* Value
LVEF, % ± SD	50 ± 7	49 ± 8	0.473
TAPSE, mm ± SD	16 ± 3	17 ± 3	0.150
S’ wave, cm/s ± SD	9.3 ± 2.4	9.5 ± 1.5	0.732
FAC, % ± SD	33 ± 8	36 ± 8	0.370
PASP, mmHg ± SD	43 ± 9.5	36.5 ± 9.1	0.034
TAPSE/PASP, mm/mmHg ± SD	0.37 ± 0.1	0.46 ± 0.1	0.011
TR grade			
1		46% (6)	0.001
2		46% (6)	
3	69% (9)		
4	23% (3)	8% (1)	
5	8% (1)		
TR VC, mm ± SD	8 ± 1	4 ± 2	<0.001
TR EROA, cm^2^ ± SD	0.63 ± 0.28	0.32 ± 0.21	<0.001
TV mean diastolic gradient, mmHg ± SD	0.9 ± 0.6	1.9 ± 1.1	0.004
TV V max, m/s ± SD	2.8 ± 0.7	2.5 ± 0.5	0.105
TR volume, mL ± SD	57 ± 16	28 ± 16	<0.001
Tricuspid annulus diameter, mm ± SD	44 ± 5	40 ± 4	<0.001
RV length, mm ± SD	60 ± 7	56 ± 6	0.08
RV middle diameter, mm ± SD	42 ± 6	35 ± 6	0.003
RV basal diameter, mm ± SD	47 ± 7	43 ± 4	0.001
RV end diastolic area, cm^2^ ± SD	19 ± 4	16 ± 3	0.049
RA area, cm^2^ ± SD	28 ± 8	26 ± 8	0.026
IVC, mm ± SD	22 ± 3	18 ± 5	0.004

EROA: effective regurgitant orifice area; FAC: fractional area change; IVC: inferior vena cava; LA: left atrium; LVEF: left ventricular ejection fraction; PASP: pulmonary artery systolic pressure; RA: right atrium; RV: right ventricle; S’ wave: right ventricular systolic myocardial velocity; TAPSE: tricuspid annular plane systolic excursion; TR: tricuspid regurgitation; TV: tricuspid valve; VC: vena contracta.

**Table 3:** Adverse events and clinical safety endpoints at follow-up.

Adverse Events and Clinical Status (N, %)	30-Day Follow-Up (N = 13)	6-Month Follow-Up (N = 10)
Cardiovascular mortality	0	0
Myocardial infarction	0	0
Stroke	0	0
New onset renal failure	0	0
Nonelective surgery for tricuspid valve repair	0	0
Endocarditis requiring surgery	0	0
Major bleeding ^1^	0	0
New onset of liver failure	0	0
Pulmonary thromboembolism	0	0
Device embolisation	0	0
Single leaflet device attachment	1 (13%)	0

^1^ One patient had a decrease in haemoglobin of 3–5 g/dL (Bleeding Academic Research Consortium classification type 3a).

## Data Availability

The data presented in this study are available on request from the corresponding author.
